# Minimal Access in Pediatric Surgery: An Overview on Progress towards Dedicated Instrument Developments and Anesthesiologic Advances to Enhance Safe Completion of Procedures

**DOI:** 10.3390/children11060679

**Published:** 2024-06-03

**Authors:** Gloria Pelizzo, Francesca Destro, Ugo Maria Pierucci, Sara Costanzo, Anna Camporesi, Veronica Diotto, Valeria Calcaterra, Amulya K. Saxena

**Affiliations:** 1 Department of Biomedical and Clinical Science, University of Milan, 20157 Milan, Italy; gloria.pelizzo@unimi.it; 2 Department of Pediatric Surgery, “V. Buzzi” Children’s Hospital, 20154 Milan, Italy; ugo.pierucci@unimi.it (U.M.P.); sara.costanzo@asst-fbf-sacco.com (S.C.); 3 Pediatric Anesthesiology and Intensive Care Unit, Department of Pediatric Surgery, “V. Buzzi” Children’s Hospital, 20154 Milan, Italy; anna.camporesi@asst-fbf-sacco.com (A.C.); veronica.diotto@asst-fbf-sacco.it (V.D.); 4 Department of Pediatrics, “V. Buzzi” Children’s Hospital, 20154 Milan, Italy; valeria.calcaterra@asst-fbf-sacco.it; 5 Department of Pediatric Surgery, Chelsea Children’s Hospital, Chelsea and Westminster Hospital NHS Fdn Trust, Imperial College London, London SW10 9NH, UK; amulya.saxena@nhs.net

**Keywords:** minimal access surgery, innovation, surgical devices, children, pediatric surgery

## Abstract

Surgical techniques are evolving in Pediatric Surgery, especially in the area of minimal access surgery (MAS) where indications for applications are expanding. Miniaturization of instruments, using natural orifices, single incisions, or remotely controlled robot-assisted procedures, promises to increase the benefits of MAS procedures in pediatrics. Many pediatric pathologies are rare, and specialized surgical and anesthesiologic instruments are necessary to manage them, defined as “orphan devices”, for which development and dissemination on the market are slowed down or sometimes hindered by regulatory standards and limiting financial conflicts of interest. In pediatric surgery, it is of utmost importance to work in a multidisciplinary way to offer a surgical path that is safe and supported by technological advances. For this reason, optimizing pediatric anesthesia is also a crucial factor where technological advances have made monitoring more precise, thereby enhancing safety in the operative room. The development of customized instruments and technologies should be supported by pediatric research and should be adapted to the individualities of the small patient. This overview outlines the importance of dedicated instruments developed for the safe completion of MAS procedures in pediatrics.

## 1. Introduction

Minimal access surgery (MAS) has undergone considerable momentum in the last three decades and can be considered a milestone for 21st-century surgery. Pediatric indications for MAS have greatly expanded with the accumulation of surgeons’ experience, the development of new devices, and the application of new technology. It is now the approach of choice for more than 80% of abdominal diseases and has been applied to practically all areas of the body [1,2,3,4,5,6]. 

Micro-instruments, the use of natural orifices, single incisions, or remotely controlled robot-assisted procedures promise to increase the benefits of surgical indications in the pediatric field [1,4,6]. Moreover, virtual reality (VR) and augmented reality (AR) are also seen as added technological advances to help manage complex malformations, tumors, and lesions requiring a tissue-sparing approach [1,6,7,8]. 

The reduced invasiveness has also involved anesthesia, where technological advances have been made to monitor patients more precisely, enhancing safety in the operative room. In pediatrics, many issues are still being discussed to support anesthesiologist procedures during MAS; these include the development of new dedicated technologies and their translation into clinical practice. 

In this overview, the instrumentation and its development, recent advancements in pediatric MAS, and present perspectives on pediatric anesthesiology have been elaborated. Factors that have hindered this development, including legislative issues (with a parallelism between the US and EU regulatory bodies) have been discussed. The importance of considering each MAS feature, starting from aspects related to the patient, surgeons, and anesthesiologists, has also been detailed in relation to crucial elements for improving results and limiting complications. 

## 2. Methods

A review was performed focusing on pediatric MAS with regard to instrument and anesthesiology advances in the pediatric age. A literature search for reviews, original papers in English, meta-analyses, and clinical trials was undertaken. All articles published in the last 10 years (2013–2023) were reviewed and selected reports specific to the pediatric age (0–18 years) were identified. Case reports were excluded, as well as case series, descriptive studies, letters, commentaries, and articles that had no full-text accessible in English. The electronic databases PubMed, Scopus, and Web of Science were searched using the following keywords (alone and/or in combination): minimal access surgery; innovation; surgical devices; pediatric anesthesiology; children; surgery; minimally invasive; laparoscopy; equipment; robotic.

## 3. Key Content and Findings

**Figure 1:**
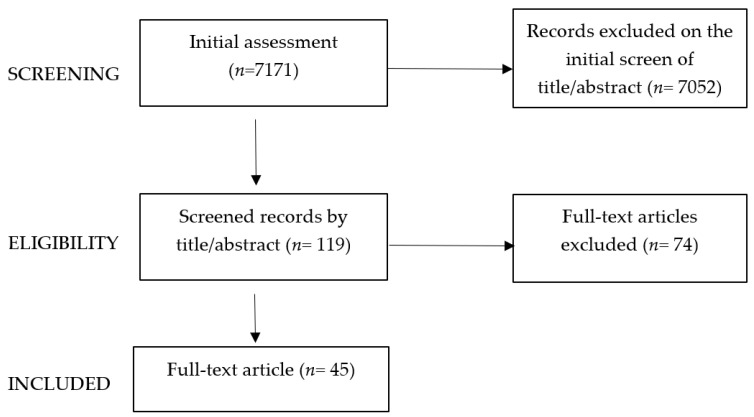
Screening and eligibility process.

The authors independently assessed abstracts and examined the full text to select the most relevant studies. Screening proceeded based on the initial assessment of papers selected by age and time of publication (n = 7171). Figure 1 resumes the screening and eligibility process. 

Eligibility included 119 papers, screened by title/abstract to exclude case reports, case series, descriptive studies, letters, commentaries, and articles not written in English. 

A total of 45 titles were identified: 14 papers on advances, innovations, and future development, 10 systematic reviews and/or meta-analyses, and 21 on anesthesiologic aspects. The current evidence on MAS and the development of devices customized for pediatric surgery were summarized. 

## 4. History of Pediatric MAS: Devices Development 

Although endoscopy is now usually thought of as a physical tool that allows for minimal access procedures, its precursor was the concept of MAS, a “new philosophy” for many early physicians [9]. One of the first was Hippocrates II (460–375 BC) [10], a dedicated advocate of reducing surgical procedures based on mortality risk. This idea has been refined and developed over the years and applied to modern endoscopy technology. Instruments and endoscopy practices were devised in Egypt in 1700–1600 BC [11]. 

### 4.1. Visualization and Light-Sourcing [11,12,13,14,15,16,17,18,19,20,21,22,23,24,25,26,27,28]

After the Egyptians and Greeks, the Romans also started using endoscopic techniques and instruments in the first century AC. However, it took some time before the issues of visibility and accessibility were overcome to make practical surgery possible. 

Arnold de Villanova (13th century) introduced candlelight to the medical world as a means of illumination, but “artificial light” was not introduced until 1500. There were humble beginnings, with Giulio Cesare Aranzi examining the nasal cavity by directing candlelight through a glass flask filled with water.

Johann Conradi and Philip Bozzini invented the first light-transmitting device, the “Lichtleiter.” However, a French physician, Antonin Jean Desormeaux, introduced the first endoscope in 1843. 

Other developments included the “speculum urethra-mystique,” a larger conical mirror that could (a) concentrate and refocus more useful light, (b) use black ocular tubes to decrease light scattering, and (c) use heated galvanized wire to raise and lower the light source. 

The Dublin urologist Francis Cruise was the first to succeed with the stereo view concept in 1865. 

Johann von Mikulicz and Joseph Leiter of Vienna improved the optical system by incorporating a prism, and Maximilian Nitze (1877) inserted a wide-angle lens in the scope that could be fully immersed, expanding the field of view. 

Multiple attempts were made to increase the amount of focused light using liquid mixtures, which were often dangerous and at risk of fire. However, designs took a sharp turn when Edison introduced the first light bulb in 1880 with the advantage that wires did not overheat and did not require bulky tubes or water for cooling.

Further improvements were made to achieve even wider views and better resolution, such as the saline solution used by Severin Nordentoeft, the modified lens by Heinz Kalk, the indirect 45-degree angle lens by John Ruddock, and the 5 mm scope by Raoul Palmer. 

British physicist Harold Hopkins is considered the most prominent inventor and pioneer of endoscopic visualization. In 1967, he developed an optical system that used “large rod-shaped quartz lenses” to improve the image projected to the eye, which is still used in modern endoscopes (Dr. Karl Storz used Palmer’s quartz rods and Hopkins’ rod lens to produce cold light and create a light source for illumination outside the body cavity). The transmissivity of quartz also dramatically improved illumination and helped decrease the sizes of endoscopes.

### 4.2. Access to Cavities (Ports) and Insufflation [15,16,17,18,19,20,21,22,23,24,25,26,27,28,29]

Trocar-like devices have existed for a long time, but it was not until 1706 that the term “trocar” was first used. The insertion of two trocars, one for visualization and the other for manipulation, occurred only in 1901. However, it should be noted that the second opening was only possible in conjunction with improvements in insufflation. 

The pioneer of artificial insufflation was the German surgeon Georg Kelling, who developed an insufflation device that filled the abdominal cavity with filtered air and recognized its safety advantages.

In 1920, Ordnoff used CO_2_, but he soon realized the possible complications (such as air embolism). A special valve needle was proposed to avoid issues from retained gas; the Veress needle is still used today, with only minor modifications, and insufflation gas monitoring was deemed an important step forward. 

The high measurement accuracy of insufflated gases obtained in 1966 by Dr Kurt Semm resulted in lower risk and safer outcomes for the patient.

## 5. MAS in Pediatrics: Recent Advancements

Technological development and innovation favored the dissemination of pediatric MAS [1,2,3,4,6,30,31,32]. Although there have been many technological advances, development times in the pediatric field are protracted compared to the adult ones [33,34]. Device development is of uttermost importance in clinical practice to overcome limitations encountered by pediatric surgeons facing accurate diagnosis and efficient treatments [33]. Initially, the problem seemed to be just a matter of size. The off-label utilization of adult-designed technology for pediatric applications was and is still frequent. Current standard-of-care practices in pediatric medicine often involve using adult-designed technologies for pediatric applications, sometimes despite the lack of more rigorous evidence [33]. It is now clear that the child is more than just a “small adult”. The efforts of creating “small instruments for children” were added in having to produce “miniaturized adult instruments”. There are unique issues when dealing with pediatric MAS. Pediatric surgical pathologies have unique and peculiar characteristics and vary in the different age groups, determining different spectrums (Table 1 shows the wide definition of “pediatric patients”) [33,34,35]. 

A child’s physiology and response to illness and stress change during growth [36]. Patient management cannot ignore the combined action of several specialists dedicated to childcare: dealing with a child means considering his complete well-being (physical, mental, and psychological). Moreover, unlike adults, children always require sedation or anesthesia during MAS, needing specific devices to cope with their peculiar anatomy and physiology [33,36,37]. Understanding this complex regulatory network sheds light on the pathogenesis of pediatric diseases and helps promote new devices and approaches for their management. 

Still, the question of the development of pediatric devices is very delicate. Over the last ten years, only 24% of Food and Drug Administration (FDA)-approved life-saving devices also have a pediatric indication [38]. FDA is a regulatory body that applies a thorough and scientific method to regulate the introduction of new devices in the United States (US). In the case of children, the FDA requires approval for medical devices associated with high risk in their use (class III, e.g., those supporting vital functions) [39]. Alternative pathways include the “investigational device exemption” (the use of a non-approved device is permitted in a clinical study with proper approval, patients’ information, and monitoring) or the “humanitarian device exemption” (humanitarian use for rare diseases, after proving the lack of additional risks for the patient). According to the FDA, there is a need for pediatric medical devices specifically designed to accommodate the unique physiology and anatomy of pediatric patients [39]. Unfortunately, from an economic point of view, investing in the development of pediatric devices is a disadvantage, as the number of children requiring devices is smaller than that of adults. Furthermore, children are highly active and subject to sudden growth changes, making the development process more difficult [33,34,36]. Clinical trials are also more difficult and can be performed only in selected hospitals. Therefore, the market discourages pediatric surgical device development [33,34,36,39]. 

In the US, the awareness of these issues has led to the drafting of the Pediatric Medical Device Safety and Improvement Act (PMDSIA), which tracks pediatric devices and facilitates their development and approval [40]. Most pediatric devices approved since the legislative change have had limited premarket studies in children, with pediatric patients representing 10% of trial participants [41]. An established academic model where pediatric surgeons work in teams with other colleagues, students, biomedicals, and engineers was described by Sack et al. [33], from identifying clinical needs and brainstorming leads to ideas and solutions, prototype development, and testing. The need for such plans arises from advancing pediatric device projects through the standard market-based grant. Including students and universities has educational implications and saves resources [33,40,41,42,43]. 

The European Union (EU) faces similar problems. The market determines low sales of pediatric devices and a reduced investment return, discouraging their development, especially with increased regulatory requirements and longer approval times. The Medical Device Regulation (MDR) applies to all EU state members. It defines the need for each new device to respect essential national requirements; stronger quality evidence is needed for high-risk devices [44]. In contrast, it does not provide special pathways for orphan medical devices (an orphan disease is defined as occurring in <1/2000 people), which can apply to most pediatric surgical pathologies [44]. Orphan/pediatric devices may be adapted from existing adult devices or developed as new. In both situations, legislative impediments could lead to renunciations in carrying out pediatric projects. Manufacturers prefer to discontinue pediatric devices and prioritize more profitable products. Compared to the US FDA, the EU MDR has a gap in the cost of assessment of around EUR 132.814 higher and a duration of assessment of around 17–23 months longer [44]. It is evident how surgery and technology are deeply influenced by each other and how the variability of effects is created by technological competitors or improvements of a leading industry. The device development process has significant challenges whose solution could derive from the interdisciplinary approach of the development cycle and clinical trial implementation. Deep and constant brainstorming leads to a better understanding of devices, identifying the best application fields, and selecting patients to refer them to. The development process usually starts at the preclinical level and passes through the translation pathway to the clinical trial [36,37]. Still, surgical changes are less controlled and occur irrepressibly [37,45]. 

### 5.1. Orphan Pediatric Devices

The incidence of pediatric diseases is minimal compared to adult diseases. This leads to many disorders in children being considered orphans, for which specific devices are therefore indispensable. Consequently, the development of pediatric surgical devices derives from those specifically prepared for adults. Pediatric surgeons often apply adult instrumentation to their population [46]. An oft-cited case in point is the surgical staplers, approved for children but not designed for them. Furthermore, production of adult devices is discontinued when viability reduces, even if they have extended pediatric application, as happened with the Linvatec arthroscopy knife disposable (CONMED, Utica, NY, USA) for laparoscopic pyloromyotomy. The process for commercial availability of products can be long and expensive. This is compounded by limited resources for orphan devices, but orphan devices are often subject to the same long and problematic regulatory process as any other product. Attention to this dilemma is growing, and there is hope for future innovation based on new resources to facilitate this process. However, the problem significantly impacts how we treat pediatric patients and needs a solution [47].

### 5.2. Equipment in Pediatric MAS

Pediatric MAS equipment (Table 2, Table 3, Table 4, Table 5 and Table 6) is designed to safely access the child’s small body, allow a good view, and permit efficient maneuvers in proper working space [1].

The systems of view have been implemented regarding dimensions and type of vision. While 10 mm diameter scopes and 5 mm instruments are used in older children and adolescents, 3 mm and 2 mm instruments with short lengths are now available for neonates and infants (by Karl Storz, Tuttlingen, Baden-Württemberg, Germany; Wolff, Knittlingen/Germany, Aesculap-B.Braun, Tuttlingen, Baden-Württemberg, Germany). Their application in advanced neonatal procedures has been established, although they are delicate tools with possible bending and grasping difficulties [1,48]. With the reduction in the size of instruments, a fundamental step for obtaining surgical success was the introduction of devices for hemostasis. Currently, 5 mm clip and stapler and dissection or sealing devices, such as the LigaSure^TM^ (Medtronic, Minneapolis, MN, USA), the JustRight (Hologic, Marlborough, Massachusetts, USA), and the En-Seal^®^ (Ethicon, Raritan, NJ, USA), are available [49,50,51]. Disadvantages of classical MAS include the rigidity of straight instrumentation, the consequent need to obtain specific angles for proper access and action, and the two-dimensional view [1]. New scopes (e.g., 4 mm Karl Storz IMAGE1 STM 3D, available with 0 and 30-degree optics) allow the reproduction of high-definition (HD) images and three-dimensional (3D) view, favoring an immersive experience in small working spaces [52]. Articulating instruments, such as the FlexDex needle driver, have been proposed to the market, allowing the same wristed angulation as the DaVinci robotic system. However, high costs still limit its use [53]. The same considerations apply to several other articulating instruments/prototypes (e.g., Radius surgical system for improved maneuverability, Artisential laparoscopic system (Livsmed, SanDiego, CA, USA) with high articulation, Hand-X 5 mm wristed electronic needle driver (by Human Xtensions, Metanya, Israel) [54,55].

## 6. Pediatric Endoscopic Surgery and Robotics

Since the 1990s, when the first laparoscopic pyloromyotomy was described, there has been increasing interest and acceptance of laparoscopy in children (Table 7) [1,5]. Mentioned advantages of the technique include better cosmesis, shorter recovery, less trauma, and better visualization [56,57]. The feasibility and safety of laparoscopy seem to be confirmed when MAS is performed by experienced surgeons [58,59]. However, recent studies have pointed out the need for more relevant research to obtain strong evidence of the real advantages of pediatric laparoscopy [1,5]. Considering the 50 most cited articles on pediatric MAS, Shu et al. detected 78% of retrospective studies and case reports with reduced scientific value compared to prospective and randomized controlled trials (RCTs) [5]. 

The diffusion of laparoscopy depends on the possibility of performing the technique in as many patients as possible (with faster learning curve improvements) and, therefore, on the incidence of the diseases, the availability of advanced laparoscopic instrumentation, and the procedure’s safety. Joint efforts are needed to implement knowledge, develop training models for rare pathologies, and foster new technologies to improve safety and simplify techniques. Robotic surgery represents another recent technological innovation that has received widespread use in adults. It allows magnified 3D visualization and improved dexterity and ergonomics [58]. High costs and the size of available instrumentation (e.g., 3 mm or smaller instruments with a shorter distance between the ports) make it difficult to apply in the pediatric age, especially in neonates and infants. Indications are still debated [58] due to the lack of strong scientific evidence (Table 8). 

Robotic surgery certainly deserves our attention. Indeed, it is supported by technological innovations that promise to provide instrumentation dedicated to pediatric patients. Moreover, training programs/VR simulators have been implemented to increase surgical competence [58]. All these elements will contribute to the diffusion of this technology in pediatric use.

## 7. Pediatric Anesthesia during MAS

The development of MAS in pediatric care goes alongside the need for anesthesia to safely allow and create the best operating conditions and to monitor such procedures. MAS causes less postoperative pain, and this has led pediatric anesthesiologists towards “less invasive” analgesic techniques. This has resulted in a decreasing use of neuraxial blocks and a growing popularity of newer US-guided blocks such as TAP and ESP. For safe performance, the use of specific equipment is essential. Industries now produce pediatric sizes of needles and catheters suitable for smaller children.

Pediatric patients, in particular premature infants, ex-premature infants, and full-term neonates, can be vulnerable to physiological changes in oxygenation and hemodynamics—which are, in the end, the two factors concurring to adequate brain preservation—that occur during anesthesia and surgery due to the pharmacologic impact of anesthetics and surgical manipulation. Recent advances in technology allow us to better control any phase of surgery from both points of view and to control the real-time effect of all on the brain. 

Ventilation of the patient during surgery is made possible by extremely precise ventilators, comparable to those used in intensive care, allowing measurement of all respiratory mechanics. Moreover, non-invasive devices, such as Electric Impedance Tomography, can show the instant distribution of ventilation in the lung and permit ventilation to adjust consequently and better set respiratory parameters. Ultrasound is also virtually ubiquitous in pediatric ORs and can be used pre-, intra-, and post-operatively to detect atelectasis at the bedside [60]. Endobronchial blockers are available in sizes that fit a few-month-old patient, allowing safe single lung ventilation in minimally invasive thoracic surgery [61]. Continuous monitoring of neuromuscular relaxation allows surgery and successive extubation with minimal risk for postoperative pulmonary complications [62]. From the hemodynamic point of view, the most common perioperative pediatric cardiac output monitoring uses surrogates such as mixed venous oxygen saturation, lactate levels, regional venous oxygen saturation, toe–core temperature difference, and serial echocardiographic exams. Non-invasive technology is becoming more popular: non-invasive monitors of perfusion (SpO2 probes with sophisticated features) can give information on the Perfusion Index, which combines information from both the hemodynamic as well the nociceptive side; regional oximeters [63], bioreactance, and impedance cardiography are currently being evaluated [64] but are still not available for extensive use [65]. Transcranial Doppler (TCD) has become a basic skill for the anesthesiologist/intensivist, allowing the detection of cerebral flow variations at the bedside with no employment of radiation.

**Figure 2:**
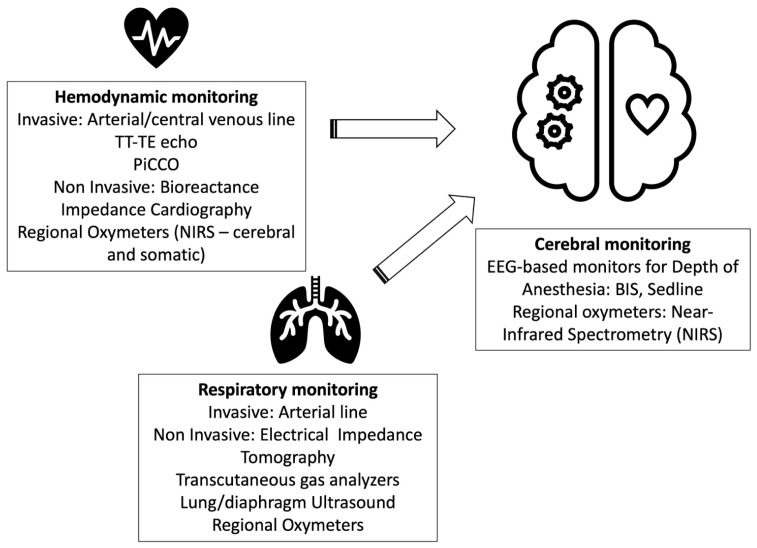
Devices used in pediatric anesthesia to monitor respiratory, hemodynamic, and cerebral functions during surgery.

Regional cerebral oxygenation can be monitored through Near Infrared Spectroscopy (NIRS), a technology that gives instant information needed for safeguarding brain function during surgery [66]. Also, cerebral activity during anesthesia and surgery is commonly evaluated with sedation monitors (BIS or Sedline, to cite some), which can show the presence of different waves instant by instant, allowing the titration of drugs to achieve the desired effects and reduce adverse events (delirium, for instance) [67] (Figure 2).

### 7.1. Cerebral Oxygenation: NIRS

Despite the diffusion of MIS, there might be concerns regarding its application on a large scale, regardless of age, type of patient, and pathology [68,69]. This reluctance arises from the small amount of information on intraoperative hemodynamic and respiratory consequences of MIS and the poor quality of evidence on MIS advantages. Recent studies have highlighted the effects of peritoneal CO_2_ insufflation on the pediatric body [68,69,70]. Interesting data come from applying NIRS to test changes in cerebral oxygenation during laparoscopy. Intraperitoneal insufflation of CO_2_ may reduce cardiac output by 15–30% by generating an elevated intra-abdominal pressure (IAP > 12–15 mmHg) that decreases venous return [68]. Another influencer of the hemodynamic effect is the arterial CO_2_ level, which increases through peritoneal absorption and blood passage of CO_2_, especially in small patients with a high peritoneal surface-to-weight ratio. The effect, mediated by the sympathetic system, is increased heart rate and contractility. It is recommended that infants and small children are monitored during surgery and in the immediate postoperative period. Pelizzo et al. showed a significant change in cerebral oxygenation (average 3.5% reduction) during traditional abdominal inguinal hernia repair [68]. The cerebrovascular system adaptation is well-known during laparoscopy. Still, studies in children are limited because results vary (some authors report no influence of CO_2_ insufflation on brain oxygenation, while others indicate various degrees of reduction) [68,71,72].

### 7.2. Hypothermia

Intraoperative hypothermia is a drop in core body temperature to below 36 °C [73]. If heat-maintaining measures are not applied, the temperature of most patients drops by 1–2 °C during surgery, a process that sets in as the intrinsic thermoregulation ceases to function [74]. Because of general anesthesia, the body cannot counteract the temperature drop by shivering and vasoconstriction. The operating room temperature is often low. However, in pediatric cases, these are adjusted as much as possible depending on the patient’s age. Laparoscopic procedures in the pediatric age group encompass patients that range from premature infants to adolescents. Though room temperatures are increased and the skin incisions in laparoscopy are much smaller than in open surgical procedures, the entire internal abdominal body surface encounters cold and dry insufflated gas (CO_2_) [75]. Hence, temperature drops during laparoscopic procedures are not much different from those during open procedures, although the size of the incision and wounds are smaller. Intraoperative hypothermia should not be underestimated as even in the mild form, it can significantly increase morbidity and mortality [72]. The unfavorable effects of intraoperative hypothermia may comprise blood coagulation disorders with increased blood loss, increased transfusion rate, myocardial dysfunction, arrhythmia, and hypokalemia [76]. This has also been implicated in delayed wound healing and infections [76,77,78].

When performing endoscopic procedures (thoracoscopy and laparoscopy), CO_2_, which is at room temperature, is usually utilized for insufflation. The CO_2_ from tanks or wall supply in the operating room is bone dry (0% relative humidity) and very cold (~20 °C, 68 F), which is ~16 °C lower than the body temperature. Studies have shown that the risk of hypothermia can be reduced by using warmed and humidified insufflation gas [79,80,81]. To quantify this, we used an infrared thermography camera in pediatrics where non-invasive monitoring of the body surface temperature could be recorded while using the HumiGard system (HumiGard, Fisher, and Paykel Healthcare). The finding demonstrated the rise in temperatures when humidified and heated CO_2_ was used during the laparoscopic procedures. Ongoing research to identify the efficacy of humidified and warmed CO_2_ is ongoing, and data from these will be important to decide on its mandatory use in all laparoscopic procedures [75,76,77,78,79].

## 8. Discussion

MAS represents an enormous resource in the pediatric surgical field. Innovation is critical for pediatric surgical advancements, specifically safer and more effective approaches [82,83,84,85]. It is a driving force for new studies but brings additional considerations and critical issues [36]. In fact, the criticalities of the pediatric age are linked to the frail condition of the child who depends on parental judgment and contingent situations. This situation inevitably leads to difficult ethical issues, especially when medical and surgical trials are involved [82,85]. Innovative therapies should be distinguished from research, outlining a proper transition from innovation to research [83,86]. Medical innovation and research are different but strictly connected. Innovation means introducing something new, while research implies study and analysis [83]. In surgery, innovation is a new technology/device or surgical technique. Research tests a hypothesis based on a formal protocol to reach an objective and increase knowledge through defined procedures [87]. According to Schwartz et al., the categories of interventions fit into “the surgical innovation continuum”, which includes three categories defined by the deviation from the current clinical practice and expected results (practice variation, transition zone, and experimental research) [85]. Practice variation can be performed without additional oversight, a slight variation from the standard approach. All experimental research is governed by the IRBs (institutional review board), research ethics, and regulatory authorities [85]. Transition (gray) zone innovations should be approached cautiously, not being experimental, although they involve more than a minor deviation from the standards. Different models for approaching transition zone innovations have been proposed (e.g., Reitsma and Moreno, Ethical, etc.) to improve a specific patient’s care [88]. These models can also be applied to implement novel devices. The introduction in clinical surgical practice of VR and AR is spreading in abdominal and thoracic MAS. Preoperative VR head-mounted display navigation in adults is reported to allow surgical strategy changes in almost 40% of the cases as a modality to prevent errors [1,6]. The application of VR in children seems to allow preoperative prevention and management of surgical risks in complex malformations, renal tumors requiring a tissue-sparing approach, and combined laparoscopic-endoscopic techniques such as biliary tree exploration congenital malformations [7,8].

We are morally called to pursue innovation in pediatric surgery to improve treatment and outcomes. Promoting innovation should imply the support of appropriate studies based on safety, protection, and regulation and limiting financial conflicts of interest [83,85,86,88]. Leaders are required to mobilize creativity, exchange ideas, and support research and innovation through the involvement of several disciplines. They should always drive investigators to ask scientific questions and carefully explore their specialty fields. Active participation in research and innovative projects is important to understand their potentialities and applications.

Timely feedback is required to identify inevitable failures in the early steps, apply corrective maneuvers, and learn to reward long-term successes [45]. Research based on solid pathways protects patients and encourages and maintains surgical knowledge [45,83]. An important step of the research method is assessing results by critically revising the hard data collected. The generation of hard data is possible through randomized, prospective, and double-blinded studies [1,6]. Considering these elements, the available literature shows little evidence of the advantages of pediatric MAS. Shu et al. show that research on pediatric MAS has increased in recent decades and is predominantly based on retrospective trials and case reports [5]. Schukfeh et al. recently presented data on meta-analyses for different pediatric MAS (Table 9, Table 10 and Table 11) [2]. MAS offers several advantages, specifically reduced enteral feedings and hospital stay time. However, perioperative complication rates and long-term sequelae require more insight. Pediatric surgeons should provide more hard data and draw considerations upon them [1,2,6].

Our paper exhibits numerous strengths alongside a few limitations. It integrates findings from diverse studies to provide a comprehensive overview of the instrumentation and evolution of MAS in pediatric surgery, offering a thorough qualitative synthesis of existing literature. It delineates recent advancements in pediatric MAS and explores perspectives on pediatric anesthesiology. Moreover, it delves into barriers to progress, such as regulatory hurdles, drawing comparisons between regulatory bodies in the US and EU. This comparative analysis likely illuminates the complexities impeding the advancement of pediatric MAS. 

Like all overviews, this paper allows for a flexible approach to synthesizing information, enabling the inclusion of a broad range of sources and perspectives without the constraints of strict methodological criteria. It furnishes rich contextual information, discussing the historical background, theoretical frameworks, and broader implications of the research. Additionally, it adeptly identifies gaps in the existing literature and proposes new areas for future research, providing a roadmap for further investigation. 

Nonetheless, due to the reliance on the author’s interpretation and synthesis of the literature, this overview is vulnerable to bias and subjectivity. Authors may unintentionally favor studies that align with their preconceptions or overlook conflicting evidence. Furthermore, its capacity to offer statistical summaries or pooled estimates of effect sizes is constrained by the inability to quantitatively synthesize data (e.g., meta-analysis). Despite these limitations, we endeavor to mitigate bias through transparent and rigorous descriptions of the available literature to offer valuable insights into the evolution of MAS in pediatric surgery.

## 9. Conclusions

This overview outlines the importance of dedicated instruments for the safer completion of MAS procedures for pediatric surgical diseases. The innovative technological advances and their integration with preoperative systems are needed to optimize MAS outcomes in children. Clinicians and researchers must work in multidisciplinary teams to bring all possible resources to assure the most advanced, personalized, and safe MAS procedures. Pediatric surgeons should definitively increase randomized and prospective studies to collect hard data and ensure real advantages and good indications of MAS in pediatric surgery. 

## Figures and Tables

**Table 1:** The definition of “Pediatric patients” in the Federal Food, Drug, and Cosmetic Act (FD&C Act).

Definition at the Time of Diagnosis or Treatment	Age Range
Neonates	From birth through the first 28 days of life
Infants	From 29 days to less than 2 years
Children	From 2 years to less than 12 years
Adolescents	Aged 12 through 21 (up to but not including the 22nd birthday)

**Table 2:** Pediatric minimal access surgery (MAS): instrumentation and features.

Videoscopes	5 mm and 10 mm
	4 mm and 10 mm IMAGE1 S^TM^ 3D (HD and 3D vision; 0- and 30-degree optic) by Karl Storz
Operative instruments (by Karl Storz, Tuttlingen, Baden-Württemberg, Germany; Wolff, Knittlingen/Germany, Aesculap-B.Braun, Tuttlingen, Baden-Württemberg, Germany)	
Grasping	2 mm (fragility; tendency to bend; grasping difficulties), 3 mm, 5 mm
Hemostasis	5-mm-clip staplers
	Ligasure^TM^ (Medtronic, Minneapolis, MN, USA)
	EnSeal^®^ (Ethicon, Raritan, NJ, USA)

**Table 3:** NOTES (natural orifice transluminal endoscopic surgery) and HYBRID NOTES (NOTES + umbilical port).

Instruments for NOTES	Indications	Challenges	Advantages
ANUBIS by Karl Storz ENDOSAMURAI (endoscope with two arms on the tip + forceps channel) by Olympus, Westborough, MA, USA	▪ transluminal endoscopic surgery approach for laparoscopic Duhamel with stapler introduced through the rectum ▪ trans gastric endoscopic drainage of pancreatic pseudocysts	Instrument clashing, suboptimal exposure, and inline placement of the instruments vs. laparoscopic triangulation	Less pain, increased aesthetic result, improved post-operative QoL

**Table 4:** Robot type and features of the instrumentation.

Da Vinci by Intuitive Surgical (Sunnyvale, CA, USA) from 2001	▪ Control unit ▪ Patient side cart with four arms ▪ 3D vision ▪ 8 mm instruments with bending tip and seven degrees of freedom (endowrist) ▪ 5 mm instruments with tentacle-like continuum tool shafts (less dexterity)
Senhance (in children > 10 kg) by Transenterix (Morrisville, NC, USA) from 2020	▪ Control unit ▪ Three or four charts, each with one arm ▪ 8 mm articulating needle driver ▪ 5 mm instruments, not articulating ▪ 3 mm instruments, not articulating
Dexter (Distalmotion, Lausanne, Switzerland)	▪ Console ▪ 8 mm fully wristed instruments ▪ No camera / optic console
Robotic system (Verb Surgical- Johnson & Johnson, New Brunswick, NJ, USA) with enhanced medical data science (emerging)	
Avatera (Avateramedical, Jena, Germany)	▪ 5 mm fully wristed instruments with seven degrees of freedom
Versius robotic system (CMR Surgical, Cambridge, UK)	▪ 5 mm fully wristed instruments
Automated suturing robots (e.g., KidsArm, Ottawa, ON, Canada, STAR, Intuitive Surgical, Sunnyvale, CA, USA)	
Deployable minirobots (minirobots inserted through incisions; research)	

**Table 5:** Articulating instruments and SILS (single-incision laparoscopic surgery) devices.

Indications	Features of the Instrumentation	
SILS or laparoscopic-assisted procedures	FlexDex (Brighton, MI, USA) needle driver (wristed angulation)	
VATS (video-assisted thoracoscopic surgery)	Radius Surgical System by Tüebingen Scientific, (Tuebingen, Germany) (improved maneuverability)	
	Artisential Laparoscopic System by Livsmed (San Diego, CA, USA) (wide range of articulating instruments)	
	5 mm Hand-X electronic articulating needle driver by Human Xtensions (Metanya, Israel)	
	SymphonX Surgical Platform for SILS by Fortimedix Surgical B.V. (Geleen, The Netherlands)	15 mm trocar Four channels (5 and 3 mm)
	Spider Surgical system (Single Port Instrument Delivery Extended Reach) for SILS by Transenterix-Asensus (Morrisville, NC, USA)	
	MUSA (robot for open microsurgical procedures) by Microsure (Son, The Netherlands)	

**Table 6:** Other MAS instrumentation.

Indications	Features of the Instrumentation
Hybrid Procedures	LECS (laparoscopic endoscopic cooperative surgery) for upper gastrointestinal tract ePARP (endoscopic assisted posterior anorectoplasty: endoscopic identification of the lower rectal pouch, endoscopic assisted transperineal puncture and dilation of the new rectal tract and modified pull-through) Combination of SILS / NOTES with robotics (robotic-assisted single port / NOTES)

**Table 7:** Topics of the most cited articles on pediatric laparoscopy.

Area	Topic	Specific Considerations
Gastrointestinal surgery	Inguinal hernia repair (1/3 of citations)	▪ Laparoscopy has advantages in case of bilateral hernia repair, incarceration and recurrence
	Appendectomy	
	Pyloromyotomy	
Urology	Pyeloplasty	▪ Safety and efficacy compared to open surgery ▪ Challenging surgical technique
	Nephrectomy	
	Ureteral reimplantation	
Thoracic surgery	Esophageal atresia	▪ Low incidence ▪ High surgical skills required ▪ Increased obstacles (e.g., limited working space, demanding anesthesia, specialized instrumentation)
	Congenital diaphragmatic hernia	
	Congenital lung malformations	
	Thoracic empyema	

**Table 8:** Indications for robotic-assisted pediatric surgery (RAS).

Area	Topic	Specific Considerations
Gastrointestinal surgery	Fundoplication	▪ No differences in the outcomes but lack of long-term follow-up ▪ Higher costs
	Other: splenectomy, Heller’s myotomy, intestinal anastomosis, anorectal pull-through, ovarian cystectomy, salpingo-oophorectomy	▪ Only case series / reports
	Hepatobiliary diseases (choledochal cyst excision, cholecystectomy)	▪ Limited studies ▪ Not recommended for hepatobiliary tumors and biliary atresia ▪ An alternative for liver donor hepatectomy
Urology (main RAS indications)	Pyeloplasty Ureteral reimplantation (Lich-Gregoire)	▪ Concerns for children < 10 kg ▪ Success rates seem similar to conventional MAS ▪ Costs and operative times are higher for RAS vs. laparoscopy
Thoracic surgery	Robotic assisted thoracoscopic surgery (RATS)	▪ Technical difficulties, especially for neonates and children ▪ An alternative for patients > 15–20 kg
Oncological surgery		▪ A safe option only in high selected cases

**Table 9:** Advantages and disadvantages of laparoscopy: data from 24 meta-analyses.

Laparoscopy	Advantages (Number of Meta-Analyses)				Disadvantages (Number of Meta-Analyses)		
	Shorter Hospital Stay	Shorter Time to Full Feeding	Reduced Complication Rate	Other (Number of Meta-Analyses)	Longer Operative Time	Higher Recurrence Rate	Other
Adhesiolysis (1)	-	-	1	-	-	-	-
Anorectal malformation repair (1)	-	-	-	-	-	-	-
Appendectomy (5)	3/5	1/5	-	Reduced wound infection (4/5), reduced bowel obstruction (4/5)	2/5	-	Intrabdominal abscess (2/5)
Choledochal cyst resection (2)	2/2	1/2	-	Reduced intraoperative blood loss (1), reduced bowel obstruction (1)	2/2	-	-
Duodenal obstruction repair (2)	-	-	-	-	1/2	-	Increased anastomotic complications (1/2)
Fundoplication (4)	1/4	1/4	1/4	Reduced retching (1/4) and morbidity at 30 days (1/4)	3/4	2/4	-
Intussusception reduction (1)	1/1	-	-	-	-	-	-
Kasai portoenterostomy (1)	-	-	-	-	-	-	Reduced survival with native liver (1/1)
Ladd’s procedure (1)	1/1	1/1	1/1	Reduced readmissions (1/1)	-	-	Postoperative volvulus (1/1)
Pyloromyotomy (5)	3/5	4/5	1/5	-	-	1/5	Increased overall complications (1/5)
Splenectomy (1)	1/1	-	-	Reduced intraoperative blood loss (1/1)	1/1	-	-

**Table 10:** Advantages and disadvantages of MAS in urology: data from 10 meta-analyses.

Urology	Advantages (Number of Meta-Analyses)				Disadvantages (Number of Meta-Analyses)		
	Shorter Hospital Stay	Shorter Time to Full Feeding	Reduced Complication Rate	Other (Number of Meta-Analyses)	Longer Operative Time	Higher Recurrence Rate	Other
Inguinal hernia repair (4)	-	-	2/4	Reduced operative time in case of bilateral hernia (3/4), reduced rate of contralateral hernia (2/4)	-	-	-
Orchidopexy (1)	1/1	-	-	-	-	-	-
Pyeloplasty (3)	2/3	-	1/3	-	2/3	-	-
Varicocelectomy (2)	-	-	-	-	-	-	-

**Table 11:** Advantages and disadvantages of thoracoscopy: data from 10 meta-analyses.

Thoracoscopy	Advantages (Number of Meta-Analyses)				Disadvantages (Number of Meta-Analyses)		
	Shorter Hospital Stay	Shorter Time to Full Feeding	Reduced Complication Rate	Other (Number of Meta-Analyses)	Longer Operative Time	Higher Recurrence Rate	Other
Congenital diaphragmatic hernia (4)	1/4	1/4	-	Reduced postoperative mortality (3/4), reduced postoperative ventilator time (2/4)	3/4	4/4	-
Esophageal atresia repair (3)	2/3	2/3	-	Reduced postoperative ventilator time (1/3)	-	-	-
Pulmonary malformation resection (2)	2/2	-	1/2	Shorter chest-tube placement (1/2)	-	-	-
Congenital diaphragmatic hernia (4)	1/4	1/4	-	Reduced postoperative mortality (3/4), reduced postoperative ventilator time (2/4)	3/4	4/4	-

## Data Availability

No new data were created.
